# Association between HMGCR, CRP, and CETP gene polymorphisms and metabolic/inflammatory serum profile in healthy adolescents

**DOI:** 10.1186/s12967-023-04571-z

**Published:** 2023-10-13

**Authors:** Benedetta Perrone, Paola Ruffo, Giuseppina Augimeri, Diego Sisci, Maria Stefania Sinicropi, Giovanni Tripepi, Corrado Mammì, Daniela Bonofiglio, Francesca Luisa Conforti

**Affiliations:** 1 Department of Pharmacy and Health and Nutritional Sciences, University of Calabria, Rende, CS Italy; 2 Centro Sanitario, University of Calabria, Via P Bucci, Rende, CS Italy; 3 Institute of Clinical Physiology of Reggio Calabria, IFC-CNR, Reggio Calabria, Italy; 4 Great Metropolitan Hospital BMM, Reggio Calabria, Italy

**Keywords:** Health, SNP, Lipid profile, Inflammation, Metabolic parameters, Statin

## Abstract

**Background:**

The complex interplay between health, lifestyle and genetics represents a critical area of research for understanding and promoting human well-being. Importantly, genetics plays a key role in determining individual susceptibility to disease and response to lifestyle. The aim of the present study was to identify genetic factors related to the metabolic/inflammatory profile of adolescents providing new insights into the individual predisposition to the different effects of the substances from the environment.

**Methods:**

Association analysis of genetic variants and biochemical parameters was performed in a total of 77 healthy adolescents recruited in the context of the DIMENU study.

**Results:**

Polymorphisms of 3-hydroxy-3-methylglutaril coenzyme A reductase (*HMGCR*; rs142563098), C-reactive protein gene (*CRP*; rs1417938, rs1130864), cholesteryl ester transfer protein (*CETP*; rs5030708), interleukin (*IL)—10* (IL-10; rs3024509) genes were significantly associated (p < 0.05) with various serum metabolic parameters. Of particular interest were also the correlations between the *HMGCR*polymorphism (rs3846663) and tumor necrosis factor (TNF)-α levels, as well Fatty-acid desaturase (*FADS*) polymorphism (rs7481842) and IL-10 level opening a new link between lipidic metabolism genes and inflammation.

**Conclusion:**

In this study, we highlighted associations between single nucleotide polymorphisms (SNPs) and serum levels of metabolic and inflammatory parameters in healthy young individuals, suggesting the importance of genetic profiling in the prevention and management of chronic disease.

## Introduction

The immune system plays a vital role in our body's response to infection, injury, and disease. Therefore, inflammation is an essential immune response that can be activated when the body detects the presence of pathogens or tissue damage. While inflammation is a protective and necessary mechanism for healing, a persistent or low-grade inflammatory profile can contribute to the development of many chronic diseases, including cardiovascular diseases (CVDs), autoimmune disease, type 2 diabetes, and even some forms of cancer [1]. Lipid metabolism, on the other hand, plays an essential role in modulating inflammation in acute and chronic diseases [2]. There is considerable evidence that dietary and endogenous lipids possess pro- and anti-inflammatory properties, while lipoprotein profiles and composition modulate atherogenic and immunomodulatory pathways in chronic metabolic and inflammatory disorders such as obesity, cardiovascular, autoimmunity, and infectious diseases [3–5].

Recent studies have also reported that cytokines can influence blood cholesterol levels and their distribution in adipose tissue, thus contributing to the development of CVDs, obesity and other related pathologies [6].

In recent years, several studies have revealed an important link between the inflammatory profile and genetic factors. In fact, our genes can influence the body's inflammatory response, determining susceptibility to certain inflammatory diseases and the extent of the immune response [7].

Genetic factors influencing the inflammatory profile may be diverse, including genes encoding inflammatory cytokines, cytokine receptors [8], enzymes involved in the metabolism of inflammatory agents, and transcription factors [9] that regulate gene expression in the context of inflammation [10], thus contributing to individual predisposition to inflammatory diseases.

Some studies have also suggested that the combination of different single nucleotide polymorphisms (SNPs) on the genes of various inflammatory molecules, such as interleukins [11–13] as well as molecules involved in brain metabolism, such as Vascular Endothelial Growth Factor (VEGF) [14, 15], show an additive effect. Moreover, high levels of interleukin (IL) -6 (IL-6), major proinflammatory cytokines, are found in the plasma of obese subjects, and the related − 174G > C polymorphism (rs1800795) upstream of the transcription start site of the gene has been associated with insulin sensitivity and plasma triglyceride levels [16]. Furthermore, SNPs in *IL-1β* and *IL-10*, pro-inflammatory and anti-inflammatory cytokines respectively, have been reported to cooperate in many viral and infectious diseases including hepatitis B [17], influenza, and pneumonia [18–21] SNPs have also been described in *transforming growth factor beta 1* (*TGFβ1*) and heart disease [20, 21].

Of note, the *CRP*, which encodes a pentameric protein synthesized by the liver, is a sensitive marker of inflammation that is not only produced in response to pro-inflammatory cytokines such as IL-1 and IL-6, but is also present in the acute phase of inflammation [22, 23]. Likewise, genetic variants in *CRP*, rs3093068, rs1130864, rs1205, have been identified in correlation with CRP concentrations [24] and previously in ischemic and haemorrhagic stroke (+ 1059G > C, + 1444C > T, − 757A > G, − 717A > G, − 286C > T > A and + 2147C > T) [25].

Regarding the influence of genetic factors on the metabolic profile, our previous results have highlighted the importance of the role of lipoprotein lipase (*LPL)*, fibronectin type III domain containing protein 5 (*FNDC5)* and peroxisome proliferator-activated receptor gamma (*PPARγ)* gene polymorphisms as determinants of health [26]. Other studies have reported reduced levels of high-density lipoprotein cholesterol (HDL-C) and an increased risk of coronary artery disease caused by increased activity of the *CETP* gene activity [27]. Specifically, associations have been reported between two polymorphisms, rs708272 (G277A) and rs5882 (I405V), and the risk of vascular disease [28].

Similarly, SNPs of *HMGCR*, encoding the key enzyme in cholesterol homeostasis [29, 30], are also associated with lipid/lipoprotein traits (such as triglycerides, total cholesterol levels, and LDL) in different populations [31].

In this study, we used high-throughput technologies to investigate the impact of SNPs on the metabolic and inflammatory serum profile of healthy adolescents and to evaluate their potential as determinants of health.

## Methods

### Participants

A total of 77 healthy adolescents (11–14 years) were enrolled and studied, as previously described [26]. The study was conducted according to the guidelines of the Declaration of Helsinki and approved by the Ethics Committee of the University of Calabria, Italy (#5727/2018).

### Genotyping

Genomic DNA was isolated from peripheral blood leukocytes, using the Wizard Genomic DNA Purification Kit (Promega), and quantified by using the NanoDrop spectrophotometer (NanoDrop™ One/OneC Microvolume UV–Vis Spectrophotometer (Thermo Fisher Scientific).

NGS analysis was performed using a targeted panel, including genes and polymorphisms related to diet, lifestyle, and physical performance/sports, on the Ion S5 sequencer (Thermo Fisher Scientific), as previously reported [23].

### Data analysis

Sequencing data generated from NGS experiments were analysed to identify single-nucleotide variants. The Torrent Suite™ (v5.12) Software (Thermo Fisher Scientific) was used for quality and coverage analysis, alignment against the GRCh37/hg19 human reference genome, and variant calling. “Germline-Low Stringency” was set as Variant Caller Parameters and annotated variants were filtered out by the following criteria: variant quality (QUAL) < 20, genotype quality (GQ) < 5, flow read space depth (FDP) < 6 and flow space alternate allele observations (FAO) < 2 [26, 32].

Variant frequencies were compared in 1000 Genomes (https://www.broadinstitute.org/) and GnomAD (https://gnomad.broadinstitute.org/) databases. Only variants with minor allele frequency (MAF) > 0.01 in each ancestry individually were included in the comparison.

The association between significant SNPs and metabolic and inflammatory variables was assessed by linear regression analysis using Tassel 5.2.21v. Non-parametric tests (Fisher’s exact test and Chi-squared) were used to evaluate important pair correlations, also applying Bonferroni’s correction. The squared of the determination coefficient (R^2^) was calculated to estimate the proportion of the variability of dependent variable which was explained by the independent variables [33].

### Covariates

Variables were described as mean and standard deviation, median and interquartile range (continuous variables) or number and percentage (categorical variables). We searched for the strength of the association between the occurrence of polymorphisms and a number of biochemicals variables using univariate linear regression analysislogistic regression analysis. The following variables were considered: circulating levels of IL-6, IL-10, TNFα, total bilirubin, and direct bilirubin. Multivariate logistic regression was also performed, testing all variables that were significantly associated with the dependent variable in univariate logistic regression. Linear regression models were used to estimate the regression coefficient (i.e., the mean increase in the dependent variable provided by each SNP) and 95% confidence intervals (CI) regarding the association independent and dependent variables. The Mann–Whitney test was used to identify differences in clinical parameters between cases and controls. All analyses were performed with the Statistical Package for Social Science (SPSS), version 24. A p–value ≤ 0.05 was considered statistically significant.

### RegulomeDB analysis

To investigate a functional context for variants or regions of interest, particularly relevant for polymorphisms located in non-coding regions, we used RegulomeDB (https://regulomedb.org/regulome-search/). This is a software based on the system of prioritization of functional SNPs identifying their presence in a DNAase hypersensitive site or a transcription factor binding site. The SNP that showed the strongest evidence of being regulatory was assigned a score of 1 and, the SNP that displayed the least evidence of being functional was marked as 6.

## Results

### NGS

We used a 13-gene NGS-based targeted resequencing. We detected 175 variants in 10 genes of interest (*IL-10, IL-6, IL-1β, TNFα, CETP, HMGCR, TGFB1, CRP, homeostatic iron regulator—(HFE), FADS*). The filtering of annotated variants was previously reported in our paper [26]. In line with the aim of this study, to identify variants with high frequency in the population, variants with MAF < 0.01 were filtered out.

Twenty-nine variants were detected, as shown in Table 1. Of these, 18 variants were located in the intronic region, 1 in the intergenic region and 7 in the flanking regions (6 in the 3’_UTR and 1 in the upstream region). The remaining 3 variants were exonic (1 synonymous and 2 missense substitutions).

**Table 1:** SNPs identified in targeted genes

Name/Gene ID	Description	Chromosomal location	SNPs	GnomAD/1000Genome	Ref Allele	Alt Allele	Location
*IL-10*	*Interleukin 10*	1:206,767,602–206,774,541	rs1518111 rs1554286 rs3024509	0.29/0.42 0.26/0.40 0.04/0.02	T A A	C G/T G	Intron variant Intron variant Intron variant
*IL-6*	*Interleukin 6*	7:22,766,819–22,771,617	rs1800795	0.29/0.14	C	G/T	Intron variant
*IL-1β*	*Interleukin 1 beta*	2:112,829,751–112,836,816	rs1071676 rs1143627 rs1143633 rs1143634 rs1143639	0.19/0.13 0.44/0.47 0.30/0.31 0.19/0.13 0.19/0.13	C G C G C	G A A/G/T A T	3_Prime UTR variant Upstream variant Intron variant Exonic variant Intron variant
*TNF-α*	*Tumor Necrosis Factor-Alpha*	6:31,575,565–31,578,336	rs3093662 rs3093664 rs3093665	0.07/0.07 0.07/0.07 0.02/0.02	A A A	G G C	Intron variant Intron variant 3_Prime UTR variant
*CETP*	*Cholesteryl Ester Transfer Protein*	16:56,961,923–56,983,845	rs708272 rs5030708	0.38/0.37 0.02/0.01	G C	A/C T	Intron variant Intron variant
*HMGCR*	*3-Hydroxy-3-Methylglutaryl-CoA Reductase*	5:75,336,329–75,364,001	rs10474435 rs10515198 rs11742194 rs12916 rs142563098 rs3846662 rs3846663 rs5909	0.01/0.01 0.08/0.07 0.08/0.07 0.36/0.41 -/0.01 0.42/0.37 0.34/0.40 0.08/0.07	T G C T T A C G	C A T A/C/G A/C G/T T A	3_Prime UTR variant Intron variant Intron variant 3_Prime UTR variant Intron variant Intron variant Intron variant 3_Prime UTR variant
*TGFB1*	*Transforming growth factor beta 1*	19:41,330,323–41,353,922	rs1800471 rs1800472 rs8179181	0.08/0.04 0.02/0.01 - / 0.07	C G G	G/T A A/C/T	Exonic variant Intron variant Exonic variant
*CRP*	*C-Reactive Protein*	1:159,682,079–159,684,379	rs1130864 rs1205 rs1417938	0.26/0.20 0.30/0.33 0.25/0.19	G C T	A T A/C	Intron variant 3_Prime UTR variant Intron variant
*FADS*	*Fatty Acid Desaturase*	11:61,799,627–61,829,318	rs7481842	0.13/0.09	C	G/T	Intergenic variant

Key: Chr: chromosome; Ref: reference; Alt: alternative

### Linear regression analysis

**Fig. 1:**
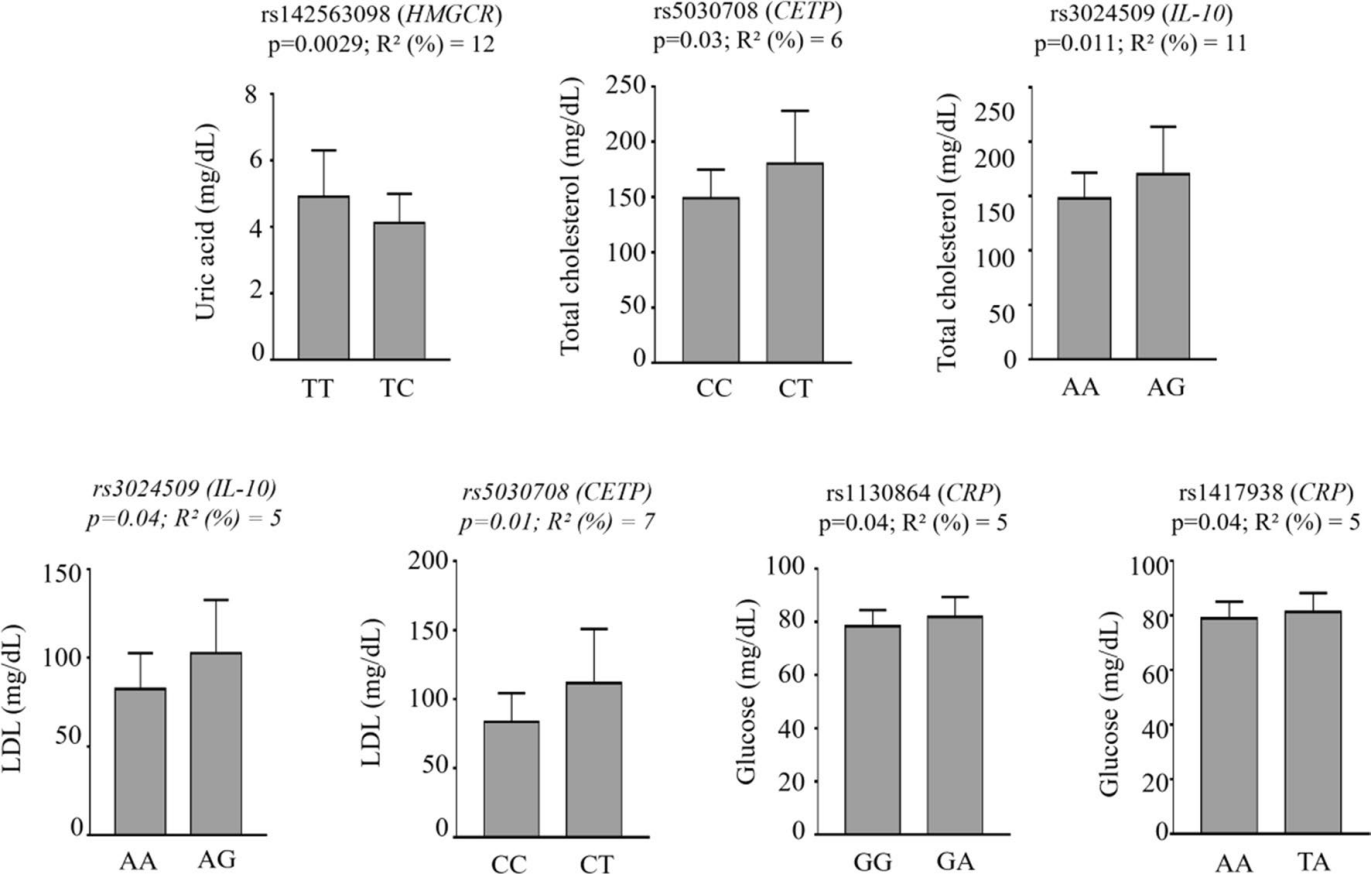
Significantly associated SNPs with inflammatory markers in our cohort. The X-axis indicates the genotype status. R2: is the statistic used for association analyses and p is the Benjamini–Hochberg adjusted p-value

By the linear regression analysis, we evaluated the correlation between the identified SNPs and the levels of inflammatory markers and found 7 significant associations (P < 0.01 and/or P < 0.05) after Bonferroni corrections. Figure 1 shows the significant associations between SNPs and parameters related to inflammatory and lipid profiles.

Our analyses showed a statistical difference in the mean uric acid levels between the two allelic subgroups in HMGCR. In particular, the rs142563098-TC genotype showed a significantly lower acid uric level (22.5 ± 3.10 mg/dl) than the TT genotype group (22.5 ± 3.10 mg/dl). Of note, after data adjustment for age, sex and cat.BMI, a positive association was observed between TNF-α levels and HMGCR/rs3846663-CT genotype, and increased levels of IL-10 in the presence of FADS/rs7481842-CG genotype (Table 2). We list the odds ratios derived from the individual analysis along with their 95% CIs and corresponding p-values for the 2 SNPs.

**Table 2:** Associations of polymorphisms in the *HMGCR* and *FADS* genes with TNF-α and IL10 levels

Gene	SNP ID	Minor Allele	Risk genotype	Serum concentration effect	*Mean increase	(95% CI)	*p*-value
*HMGCR*	rs3846663	T	CT	**↑**TNF-α	+ 0.49	0.05–0.92	0.033
*FADS*	rs7481842	C	CG	**↑**IL-10	+ 1.09	0.34–1.85	0.006

SNP, Single Nucleotide polymorphism; CI, confidence interval.

*Derived from the slope of the linear regression analysis

In addition, the IL-10/rs3024509-AG genotype was associated with increased low-density lipoprotein (LDL) (82 ± 20 vs 100 ± 29) and total cholesterol (147 ± 23 vs 170 ± 43) concentrations; however, we did not observe the same difference after adjustment for sex, age and cat.BMI. Furthermore, cholesterol and LDL levels were significantly increased in the presence of the CETP/rs5030708-CT genotype (180 ± 47 vs 149 ± 25 and 111.75 ± 39 vs 83.6 ± 20.7). Likewise, CRP/rs1417938-TA and CRP/rs1130864-GA genotypes were significantly correlated with higher fasting glucose levels (81.6 ± 7.26 vs 78.9 ± 5.89 and 81.25 ± 7.17 vs 78.9 ± 5.87, respectively).

In order to understand whether the identified polymorphisms in non-coding regions have functional consequences, we evaluated their impact through RegulomeDB ranks. Data retrieved from this software showed that most of the identified SNPs have a probable regulatory role, with a ranking between 1 and 5 for all except one (Table 3).

**Table 3:** List of the non-coding SNPs and related RegulomeDB scores

dbSNP IDs	Chromosome location	Rank	Score
rs3846663	chr5:74655725–74655726	1f	0.55324
rs3024509	chr1:206943296–206943297	3a	0.55134
rs5030708	chr16:56996278–56996279	3a	0.47027
rs142563098	chr5:74633013–74633014	4	0.60906
rs1417938	chr1:159684185–159684186	5	0.58955
rs7481842	chr11:61639704–61639705	5	0.13454
rs1130864	chr1:159683090–159683091	7	0.18412

RegulomeDb category summaries [33]. Rank refers to the supporting evidence for that particular location or variant id. In general, if more supporting data is available, the higher is its likelihood of being functional and hence receives a higher score (with 1 being higher and 7 being lower score); 1b-1f: Likely to affect binding and linked to expression of a gene target; 2a-2c: Likely to affect binding; 3a-3b: Less likely to affect binding;4-5-6: Minimal binding evidence

The RegulomeDB probability score is ranging from 0 to 1, with 1 being most likely to be a regulatory variant

## Discussion

The present study analysed the association between serum metabolic/inflammatory parameters and the occurrence of polymorphisms in a sample of healthy adolescents from Southern Italy. In detail, we have identified a new genetic polymorphism in the HMGCR gene, rs142563098 associated with acid uric. Several studies have suggested that serum uric acid is correlated with CVD, but some studies have reported contradictory results. However, recent meta-analyses of prospective studies have supported that hyperuricemia is an independent risk factor for CVDs [34–36].

It has demonstrated, also, that uric acid induces reactive oxygen species (ROS) production and it activates several intracellular signalling pathways that result in the production of inflammatory cytokines, adhesion factors, and chemokines regulating cell proliferation and apoptosis and in turn leading to atherosclerosis development [37].

Interestingly, in our cohort, we found increased serum TNF-α levels in the presence of the *HMGCR*/rs3846663 T allele (p < 0.05). This polymorphism has previously been identified in genome-wide association (GWA) studies as being associated with increased LDL-cholesterol (LDL-C) levels [31, 38]. Although the presence of the *HMGCR* variants is already known and associated with lipid profile, we have shown for the first time the involvement of SNPs of this enzyme in inflammatory status.

Statins are a well-established family of drugs that lower cholesterol levels via the competitive inhibition of the HMGCR enzyme. Statins also have anti-inflammatory effects, including reducing CRP concentrations [39]. Furthermore, statins reduce TNF-α and interferon gamma (INFγ) production in stimulated T-lymphocytes and inhibit the T helper cell (Th-1) immune response [40]. Addition of statins to human hepatocytes reduces CRP levels induced by circulating IL-6, suggesting that the anti-inflammatory effects of statins are hepatic in nature [41].

These effects of statin treatment are most likely not indirect and mediated by decreased cholesterol levels, but rather direct and could be due to decreased protein prenylation, another HMGCR-dependent reaction. Protein prenylation is a posttranslational modification of proteins, which results in the covalent modification of these proteins with the mevalonate pathway intermediates as farnesyl pyrophosphate or geranylgeranyl pyrophosphate. The lipophilic prenyl groups enable proteins to anchor to cell membranes or facilitate protein–protein interactions. Important prenylated proteins include members of the Ras superfamily of small GTPases, such as Ras and Rho, involved in the proliferation and differentiation processes of cells [42]. The RhoA–NFκB interaction has been shown to be important in cytokine-activated NFκB processes, such as those induced by TNF-α [43, 44].

We also found increased serum levels of IL-10 in the presence of the *FADS* SNPs rs7481842, while a known correlation between polymorphisms in the FADS gene and serum lipids in GWA studies has been described previously [45]. However, no associations with anthropometric measures and lipid parameters were reported in our sample, probably due to the small size of the population studied. Fatty acid desaturase 1 and 2 (*FADS1* and *FADS2*, respectively) genes encode for key enzymes in the Polyunsaturated Fatty Acid (PUFA) metabolism, the δ-5 desaturase (D5D) and D6D, respectively [46]. In populations eating a Western diet rich in omega-6 PUFA, a high desaturase activity may promote increased bioavailability of arachidonic acid with a prevailing synthesis of arachidonic acid-derived proinflammatory eicosanoids, finally favoring atherosclerotic vascular damage. In contrast, high desaturase activity in subjects consuming a diet rich in omega-3 PUFA or receiving omega-3 PUFA supplementation could result in the opposite situation with a preferential synthesis of anti-inflammatory eicosanoids. For these reasons, people carrying specific *FADS* haplotype polymorphisms may be predisposed to more pronounced vascular inflammatory damage in the context of a Western diet, but also to an increased beneficial effect of omega-3 PUFA supplementation [46]. It is therefore also important to bear in mind that diseases are caused not only by genetic factors but also by a complex combination of environmental determinants. Another novel association was observed between the *CRP*/rs1417938 TA genotype and glucose levels (p < 0.05) in our study. Moreover, we also observed the relationship between the rs1130864-GA genotype and increased fasting glucose levels (p < 0.05), as previously reported [47]. Our findings, confirming the direct correlation between this gene and glucose homeostasis, further support the influence of genetic factors in metabolic profiles related to inflammation genes.

Moreover, in our study, analysis of the rs5030708 polymorphism in *CETP* showed a statistically significant increase of total (p < 0.05) and LDL cholesterol (p ≤ 0.01) levels, which has never been reported in the literature. CETP is a glycoprotein that is synthesized in the liver and promotes bidirectional transfer of cholesteryl esters and triglycerides between all plasma lipoprotein particles: (i) transfer of cholesteryl esters from cholesteryl ester-rich HDL particles to LDL and very LDL (VLDL) particles and (ii) transfer of triglycerides from triglyceride-rich VLDL particles and chylomicrons to HDL and LDL particles. Thereby, CETP has a direct effect on both plasma HDL-C as well as LDL-C levels [48]. The *CETP* gene, located on chromosome 16q21, is highly polymorphic and polymorphisms in this gene have a differential effect on the HDL-C fraction. The importance of plasma CETP in lipoprotein metabolism was demonstrated by the discovery of CETP-deficient subjects with marked hyperalphalipoproteinaemia (HALP) [49]. When CETP is high, the efficiency of HDL to transfer triglycerides (TGs) is increased, leading to a reduction in TGs, HDL particles are rapidly cleared, and HDL-C levels are reduced [49]. Previous association studies have indicated that *CETP* polymorphisms are associated with lower HDL-C concentrations in children with a family history of diseases of the cardiovascular system [50]. Other results have shown that *CETP* SNPs interact with dietary carbohydrate intake on metabolic factors, such as hypertension, dyslipidaemia and, obesity. Specifically, a potential interaction between polymorphism in this gene and dietary fat on plasma lipid and lipoprotein concentrations has been reported [51, 52], suggesting that the CETP gene also plays a crucial role.

It is good to note that the role of non-coding variations of different genes has already been reported relating to inflammatory phenomena and several diseases. For example, IL-1B (-511C > T) has been reported to be associated with the severity and progression of multiple sclerosis (MS), while the NLRC4 rs479333 G > C variant has shown beneficial effects by limiting disease progression and supporting response to treatment with INF-β [53]. In addition, different human leukocyte antigen G (HLA-G) polymorphisms have been associated with distinct levels of HLA-G expression and with the development of sepsis. Intronic SNPs in the PTNP2 gene have been associated with changes in PTPN2 expression and modulation of binding to important transcription factors. PTPN2 protein was overexpressed in inflamed intestinal tissue of patients with Chron’s disease [54].

Overall, to the best of our knowledge, this is the first study showing novel associations between *HMGCR*, *CRP,* and *CETP* polymorphisms and serum metabolic and inflammatory parameters in healthy adolescents, indicating that these polymorphisms may act as risk factors influencing the metabolic/inflammatory profile in a young population. Disrupting the complex interplay between lipid and inflammatory profiles could be a strategy to limit the risk of the development of chronic diseases. In this context, among different molecules, statins are small inhibitors of cholesterol synthesis, able to alter proinflammatory metabolic signatures to potentially lessen these disease pathogeneses [55]. In addition to this, it should be taken into account that, in the presence of certain genetic variants, the use of these pleiotropic molecules may positively impact the metabolic/inflammatory profile modifying the risk factors of related diseases, and thus confirming the relevance of genetics in personalized medicine. Although the limitation of our study is represented by the small size of our cohort which could make it difficult to generalize the data, the present results emphasize the importance of genetic profiling as a determinant of health. Further research is needed to validate these findings in a larger population and to explore the underlying mechanisms involved.

## Data Availability

The data presented in this study are available in results.

## Abbreviations

SNPs: Single nucleotide polymorphisms
HMGCR: 3-Hydroxy-3-methylglutaryl-coenzyme A reductase
VEGF: Vascular endothelial growth factor
CRP: C-reactive protein (CRP)
CETP: Cholesteryl ester transfer protein
IL: Interleukin
TGFβ1: Transforming growth factor beta 1
HDL-C: High-density lipoprotein cholesterol
QUAL: Variant quality
GQ: Genotype quality
FDP: Flow space read depth
FAO: Flow space alternate allele observations
MAF: Minor allele frequency
GLM: General linear model
OR: Odd ratios
CI: Confidence intervals
Chr: Chromosome
LDL: Low-density lipoprotein
TNF-α: Tumor necrosis factor alpha
INFγ: Interferon gamma
HALP: Hyperalphalipoproteinaemia
TG: Trygliceride

## References

[1] Pahwa R, Goyal A, Jialal I. Chronic Inflammation. In *StatPearls*; StatPearls Publishing Copyright © 2023, StatPearls Publishing LLC.: Treasure Island (FL) ineligible companies. Disclosure: Amandeep Goyal declares no relevant financial relationships with ineligible companies. Disclosure: Ishwarlal Jialal declares no relevant financial relationships with ineligible companies. 2023;2.

[2] Tall AR, Yvan-Charvet L (2015). Cholesterol, inflammation and innate immunity. Nat Rev Immunol.

[3] Andersen CJ (2018). Impact of dietary cholesterol on the pathophysiology of infectious and autoimmune disease. Nutrients.

[4] Kumar NG, Contaifer D, Madurantakam P, Carbone S, Price ET, Van Tassell B, Brophy DF, Wijesinghe DS (2019). Dietary bioactive fatty acids as modulators of immune function: implications on human health. Nutrients.

[5] Huang J, Yancey PG, Tao H, Borja MS, Smith LE, Kon V, Davies SS, Linton MF (2020). Reactive dicarbonyl scavenging effectively reduces MPO-mediated oxidation of HDL and restores PON1 Activity. Nutrients.

[6] Chait A, den Hartigh LJ (2020). Adipose tissue distribution, inflammation and its metabolic consequences, including diabetes and cardiovascular disease. Front Cardiovascular Med.

[7] Chen L, Deng H, Cui H, Fang J, Zuo Z, Deng J, Li Y, Wang X, Zhao L (2018). Inflammatory responses and inflammation-associated diseases in organs. Oncotarget.

[8] Lio D, Licastro F, Scola L, Chiappelli M, Grimaldi LM, Crivello A, Colonna-Romano G, Candore G, Franceschi C, Caruso C (2003). Interleukin-10 promoter polymorphism in sporadic Alzheimer's disease. Genes Immun.

[9] Ding H, Liu XC, Jian-Ming X, Qiao M (2021). Identification of crucial genes and related transcription factors in ulcerative colitis. Ann Clin Lab Sci.

[10] Stoeckman AK, Baechler EC, Ortmann WA, Behrens TW, Michet CJ, Peterson EJ (2006). A distinct inflammatory gene expression profile in patients with psoriatic arthritis. Genes Immun.

[11] Bei CH, Bai H, Yu HP, Yang Y, Liang QQ, Deng YY, Tan SK, Qiu XQ (2014). Combined effects of six cytokine gene polymorphisms and SNP-SNP interactions on hepatocellular carcinoma risk in Southern Guangxi, China. Asian Pacific J Cancer Prevent APJCP.

[12] Miteva L, Stanilova S (2008). The combined effect of interleukin (IL)-10 and IL-12 polymorphisms on induced cytokine production. Hum Immunol.

[13] Scola L, Giarratana RM, Marinello V, Cancila V, Pisano C, Ruvolo G, Frati G, Lio D, Balistreri CR (2021). Polymorphisms of pro-inflammatory IL-6 and IL-1β cytokines in ascending aortic aneurysms as genetic modifiers and predictive and prognostic biomarkers. Biomolecules.

[14] Tung GK, Sambyal V, Guleria K (2022). Association of VEGF -2549 I/D and VEGF +936 C/T polymorphisms with chronic Kidney disease in North-West Indian patients. Indian J Nephrol.

[15] Yi JP, Wu YZ, Yu N, Yu ZW, Xie FY, Yuan Q (2016). VEGF gene polymorphisms affect serum protein levels and alter disease activity and synovial lesions in rheumatoid arthritis. Med Sci Monit Int Med J Exp Clin Res.

[16] Testa R, Olivieri F, Bonfigli AR, Sirolla C, Boemi M, Marchegiani F, Marra M, Cenerelli S, Antonicelli R, Dolci A (2006). Interleukin-6-174 G > C polymorphism affects the association between IL-6 plasma levels and insulin resistance in type 2 diabetic patients. Diabetes Res Clin Pract.

[17] Tunçbilek S (2014). Relationship between cytokine gene polymorphisms and chronic hepatitis B virus infection. World J Gastroenterol.

[18] Su G, Ding L, Zhang Z (2019). The effect of lnterleukin-6 gene polymorphism on pediatric pneumonia. Iran J Public Health.

[19] Gallagher PM, Lowe G, Fitzgerald T, Bella A, Greene CM, McElvaney NG, O'Neill SJ (2003). Association of IL-10 polymorphism with severity of illness in community acquired pneumonia. Thorax.

[20] Calzada JE, Beraún Y, González CI, Martín J (2009). Transforming growth factor beta 1 (TGFbeta1) gene polymorphisms and Chagas disease susceptibility in Peruvian and Colombian patients. Cytokine.

[21] Chen Y, Dawes PT, Packham JC, Mattey DL (2012). Interaction between smoking and functional polymorphism in the TGFB1 gene is associated with ischaemic heart disease and myocardial infarction in patients with rheumatoid arthritis: a cross-sectional study. Arthritis Res Ther.

[22] Enocsson H, Gullstrand B, Eloranta ML, Wetterö J, Leonard D, Rönnblom L, Bengtsson AA, Sjöwall C (2020). C-reactive protein levels in systemic lupus erythematosus are modulated by the interferon gene signature and CRP gene polymorphism rs1205. Front Immunol.

[23] Auerkari E, Suhartono A, Djamal N, Verisqa F, Suryandari D, Kusdhany L, Masulili S, Talbot C (2013). CRP and IL-1B gene polymorphisms and CRP in blood in periodontal disease. Open Dent J.

[24] Arouca A, Michels N, Moreno LA, González-Gil EM, Marcos A, Gómez S, Díaz LE, Widhalm K, Molnár D, Manios Y (2018). Associations between a Mediterranean diet pattern and inflammatory biomarkers in European adolescents. Eur J Nutr.

[25] Das S, Roy S, Kaul S, Jyothy A, Munshi A (2014). CRP gene (1059G>C) polymorphism and its plasma levels in ischemic stroke and hemorrhagic stroke in a south Indian population. Inflammation.

[26] Perrone B, Ruffo P, Zelasco S, Giordano C, Morelli C, Barone I, Catalano S, Andò S, Sisci D, Tripepi G (2022). LPL, FNDC5 and PPARγ gene polymorphisms related to body composition parameters and lipid metabolic profile in adolescents from Southern Italy. J Transl Med.

[27] Gordon DJ, Probstfield JL, Garrison RJ, Neaton JD, Castelli WP, Knoke JD, Jacobs DR, Bangdiwala S, Tyroler HA (1989). High-density lipoprotein cholesterol and cardiovascular disease. Four Prospect Am Stud Circulat.

[28] Wang Q, Zhou SB, Wang LJ, Lei MM, Wang Y, Miao C, Jin YZ (2014). Seven functional polymorphisms in the CETP gene and myocardial infarction risk: a meta-analysis and meta-regression. PLoS ONE.

[29] Shao W, Espenshade PJ (2001). Lipids: cholesterol synthesis and regulation. Encycl Biol Chem.

[30] Das KC, Hossain MU, Moniruzzaman M, Salimullah M, Akhteruzzaman S (2022). High-risk polymorphisms associated with the molecular function of human HMGCR gene infer the inhibition of cholesterol biosynthesis. Biomed Res Int.

[31] Schroor MM, Mokhtar FBA, Plat J, Mensink RP (2021). Associations between SNPs in intestinal cholesterol absorption and endogenous cholesterol synthesis genes with cholesterol metabolism. Biomedicines.

[32] Damiati E, Borsani G, Giacopuzzi E (2016). Amplicon-based semiconductor sequencing of human exomes: performance evaluation and optimization strategies. Hum Genet.

[33] Boyle AP, Hong EL, Hariharan M, Cheng Y, Schaub MA, Kasowski M, Karczewski KJ, Park J, Hitz BC, Weng S (2012). Annotation of functional variation in personal genomes using RegulomeDB. Genome Res.

[34] Zuo T, Liu X, Jiang L, Mao S, Yin X, Guo L (2016). Hyperuricemia and coronary heart disease mortality: a meta-analysis of prospective cohort studies. BMC Cardiovasc Disord.

[35] Li M, Hu X, Fan Y, Li K, Zhang X, Hou W, Tang Z (2016). Hyperuricemia and the risk for coronary heart disease morbidity and mortality a systematic review and dose-response meta-analysis. Sci Rep.

[36] Zhao G, Huang L, Song M, Song Y (2013). Baseline serum uric acid level as a predictor of cardiovascular disease related mortality and all-cause mortality: a meta-analysis of prospective studies. Atherosclerosis.

[37] Kimura Y, Tsukui D, Kono H (2021). Uric acid in inflammation and the pathogenesis of atherosclerosis. Int J Mol Sci.

[38] Kathiresan S, Willer CJ, Peloso GM, Demissie S, Musunuru K, Schadt EE, Kaplan L, Bennett D, Li Y, Tanaka T (2009). Common variants at 30 loci contribute to polygenic dyslipidemia. Nat Genet.

[39] Montecucco F, Burger F, Pelli G, Poku NK, Berlier C, Steffens S, Mach F (2009). Statins inhibit C-reactive protein-induced chemokine secretion, ICAM-1 upregulation and chemotaxis in adherent human monocytes. Rheumatology (Oxford).

[40] Link A, Ayadhi T, Böhm M, Nickenig G (2006). Rapid immunomodulation by rosuvastatin in patients with acute coronary syndrome. Eur Heart J.

[41] Mayer C, Gruber HJ, Landl EM, Pailer S, Scharnagl H, Truschnig-Wilders M, März W (2007). Rosuvastatin reduces interleukin-6-induced expression of C-reactive protein in human hepatocytes in a STAT3- and C/EBP-dependent fashion. Int J Clin Pharmacol Ther.

[42] Greenwood J, Steinman L, Zamvil SS (2006). Statin therapy and autoimmune disease: from protein prenylation to immunomodulation. Nat Rev Immunol.

[43] Tong L, Tergaonkar V (2014). Biosci Reports.

[44] Antonopoulos AS, Margaritis M, Lee R, Channon K, Antoniades C (2012). Statins as anti-inflammatory agents in atherogenesis: molecular mechanisms and lessons from the recent clinical trials. Curr Pharm Des.

[45] Malerba G, Schaeffer L, Xumerle L, Klopp N, Trabetti E, Biscuola M, Cavallari U, Galavotti R, Martinelli N, Guarini P (2008). SNPs of the FADS gene cluster are associated with polyunsaturated fatty acids in a cohort of patients with cardiovascular disease. Lipids.

[46] Martinelli N, Girelli D, Malerba G, Guarini P, Illig T, Trabetti E, Sandri M, Friso S, Pizzolo F, Schaeffer L (2008). FADS genotypes and desaturase activity estimated by the ratio of arachidonic acid to linoleic acid are associated with inflammation and coronary artery disease. Am J Clin Nutr.

[47] Kato K, Otsuka T, Saiki Y, Kobayashi N, Nakamura T, Kon Y, Kawada T (2019). Association between elevated c-reactive protein levels and prediabetes in adults, particularly impaired glucose tolerance. Can J Diabetes.

[48] Barter PJ, Hopkins GJ, Calvert GD (1982). Transfers and exchanges of esterified cholesterol between plasma lipoproteins. Biochem J.

[49] Inazu A, Brown ML, Hesler CB, Agellon LB, Koizumi J, Takata K, Maruhama Y, Mabuchi H, Tall AR (1990). Increased high-density lipoprotein levels caused by a common cholesteryl-ester transfer protein gene mutation. N Engl J Med.

[50] Pac-Kożuchowska E, Krawiec P (2013). Cholesterol ester transfer protein (CETP) gene polymorphism and selected parameters of lipid metabolism in children from families with history of cardiovascular system diseases. Med Sci Monit Int Med J Exp Clin Res.

[51] Wuni R, Kuhnle GGC, Wynn-Jones AA, Vimaleswaran KS (2022). A Nutrigenetic update on CETP gene-diet interactions on lipid-related outcomes. Curr Atheroscler Rep.

[52] Abaj F, Rafiee M, Koohdani F (2021). Interaction between CETP polymorphism and dietary insulin index and load in relation to cardiovascular risk factors in diabetic adults. Sci Rep.

[53] Soares JL, Oliveira EM, Pontillo A (2019). Variants in NLRP3 and NLRC4 inflammasome associate with susceptibility and severity of multiple sclerosis. Mult Scler Relat Disord.

[54] Marcil V, Mack DR, Kumar V, Faure C, Carlson CS, Beaulieu P, Israel D, Krupoves A, Costea I, Lambrette P (2013). Association between the PTPN2 gene and Crohn's disease: dissection of potential causal variants. Inflamm Bowel Dis.

[55] Zhong Z, Feng X, Su G, Du L, Liao W, Liu S, Li F, Zuo X, Yang P (2021). HMG-coenzyme a reductase as a drug target for the prevention of ankylosing spondylitis. Front Cell Develop Biol.

